# Visualizing subcellular rearrangements in intact β cells using soft x-ray tomography

**DOI:** 10.1126/sciadv.abc8262

**Published:** 2020-12-09

**Authors:** Kate L. White, Jitin Singla, Valentina Loconte, Jian-Hua Chen, Axel Ekman, Liping Sun, Xianjun Zhang, John Paul Francis, Angdi Li, Wen Lin, Kaylee Tseng, Gerry McDermott, Frank Alber, Andrej Sali, Carolyn Larabell, Raymond C. Stevens

**Affiliations:** 1 Department of Biological Sciences, Bridge Institute, USC Michelson Center for Convergent Bioscience, University of Southern California, Los Angeles, CA 90089, USA.; 2 Lawrence Berkeley National Laboratory, Berkeley, CA 94720, USA.; 3 Institute for Quantitative and Computational Biosciences, Department of Microbiology, Immunology, and Molecular Genetics, University of California, Los Angeles, Los Angeles, CA 90095, USA.; 4 iHuman Institute, School of Life Science and Technology, ShanghaiTech University, Shanghai 201210, China.; 5 Department of Anatomy, University of California, San Francisco, San Francisco, CA 94143, USA.; 6 Department of Computer Science, Bridge Institute, USC Michelson Center for Convergent Bioscience, University of Southern California, Los Angeles, CA 90089, USA.; 7 Department of Chemistry, Bridge Institute, USC Michelson Center for Convergent Bioscience, University of Southern California, Los Angeles, CA 90089, USA.; 8 California Institute for Quantitative Biosciences, Department of Bioengineering and Therapeutic Sciences, Department of Pharmaceutical Chemistry, University of California, San Francisco, San Francisco, CA 94158, USA.

## Abstract

Characterizing relationships between cell structures and functions requires mesoscale mapping of intact cells showing subcellular rearrangements following stimulation; however, current approaches are limited in this regard. Here, we report a unique application of soft x-ray tomography to generate three-dimensional reconstructions of whole pancreatic β cells at different time points following glucose-stimulated insulin secretion. Reconstructions following stimulation showed distinct insulin vesicle distribution patterns reflective of altered vesicle pool sizes as they travel through the secretory pathway. Our results show that glucose stimulation caused rapid changes in biochemical composition and/or density of insulin packing, increased mitochondrial volume, and closer proximity of insulin vesicles to mitochondria. Costimulation with exendin-4 (a glucagon-like peptide-1 receptor agonist) prolonged these effects and increased insulin packaging efficiency and vesicle maturation. This study provides unique perspectives on the coordinated structural reorganization and interactions of organelles that dictate cell responses.

## Teaser

Subcellular maps of β cells reveal the coordinated reorganization of organelles during insulin secretion.

## INTRODUCTION

The mesoscale architecture of a cell encompasses the organization of all materials ranging in scale from the whole single cell (~10 μm diameter) to objects just larger than molecular machines (~50 nm) (*1*). To more accurately characterize relationships between the substructures and functions of a cell, we must consider how its organelles interact, traffic, and recycle, as well as the related topological rearrangements that occur during the life span of the cell. At the mesoscale, these processes include changes in chromatin organization, organelle volumes and distributions, and locations and structural features of different cellular neighborhoods that retain specific functions. Thus, to characterize the role of subcellular structure in specific neighborhood functions, we need a comprehensive three-dimensional (3D) view of the entire cell under multiple stimulus conditions.

Over the last several decades, cryo–electron microscopy (EM), tomography, and fluorescence imaging have unveiled features of cellular structure at ever increasing resolution. However, current methodological limitations present challenges for quantifying subcellular topology rearrangements in whole, intact native cells. EM approaches require sample sectioning and often plastic embedding or chemical fixation, thereby limiting the number of cells and conditions that can be investigated in practice (*2*, *3*). Powerful advances in cryo–electron tomography have enabled near-native 3D structural investigation of specific cellular neighborhoods, providing valuable insight into sections of a cell, but not whole-cell quantification (*4*–*6*). New applications and approaches of fluorescence imaging have opened up possibilities for examining 3D cellular organization at higher resolution, including details on organelle and protein localization (*7*–*10*). However, these fluorescent microscopy approaches require the use of fluorescent probes, which limit the number and type of molecules that can be imaged at one time. These constraints hinder the ability to image specific organelles while still capturing the identities, locations, and structures of neighboring organelles or cellular components, thus limiting unbiased discovery. Therefore, with traditional methods alone, it is challenging to reconstruct 3D volumes of multiple cells under a variety of conditions, as is required for mapping the dynamic processes involved in subcellular reorganization.

Secretory cell research would benefit from the ability to map the dynamic processes of subcellular reorganization in 3D. For example, pancreatic β cells are responsible for secreting insulin in response to glucose. Glucose stimulates an initial pool of insulin secretory vesicles to fuse with the cell’s plasma membrane (PM) causing an initial phase of secretion, followed by a second phase as more vesicles move into position near the membrane (*11*). However, many of the subcellular rearrangements corresponding to glucose-stimulated insulin secretion remain unclear. The 3D topology of pancreatic β cells, i.e., the number and distributions of organelles, along with cell ultrastructure, has been characterized by EM (*3*). However, these studies were limited by laborious sample preparation, making the analysis of multiple cells under several conditions unfeasible. In a complementary effort, live cell total internal reflection fluorescence (TIRF) microscopy has been used to measure insulin vesicle fusion rates at the cell’s PM (*11*). The minimal penetration depth of TIRF limited these investigations to vesicle fusion at the membrane. While these approaches have provided critical insight into the number of organelles packed inside a single cell, as well as the timing and numbers of insulin vesicles fusing to the PM, they do not allow mapping of the 3D topology rearrangements inside whole cells during stimulation. Thus, many unanswered questions remain regarding how β cells respond to various insulinotropic stimuli, including the timing of insulin vesicle biogenesis and the path of vesicle maturation, secretion, recycling, and degradation.

To map the mesoscale topology rearrangements in a whole cell under multiple conditions, here, we present an approach using soft x-ray tomography (SXT). An advantage of SXT is that it allows observation of the near-native (i.e., cryo-fixed instead of chemically fixed), fully hydrated cellular architecture of intact whole cells (*1*). In addition, SXT datasets can be collected rapidly (<10 min per cell), thereby enabling the analysis of an increased number of samples and conditions compared to other imaging methods. Because SXT uses the inherent contrast of organic material within a sample, fluorescent probes or x-ray contrast agents are not required. Rather, cells are imaged within the water window (284 to 543 eV), where carbon-rich material has a higher linear absorption coefficient (LAC) than the surrounding background. This allows accurate and simultaneous identification of intracellular structures, such as membranes, the nucleus, nucleolus, mitochondria, endoplasmic reticulum (ER), and lipid droplets in the same cell (*1*, *12*). Our unique approach allows subcellular mapping of topological rearrangements and molecular densities of organelles (measured by LAC values) that correspond to changes in cell functions upon drug treatment (figs. S1 and S2). Acquiring this information was not possible with previously applied approaches. Our results provide insights into the 3D topological rearrangements, physical associations, and molecular densities of organelles within whole cells during glucose-stimulated insulin secretion and are expected to be invaluable for whole-cell modeling of β cells (*13*).

## RESULTS

### Mapping reorganization of organelles during insulin secretion

**Fig. 1:**
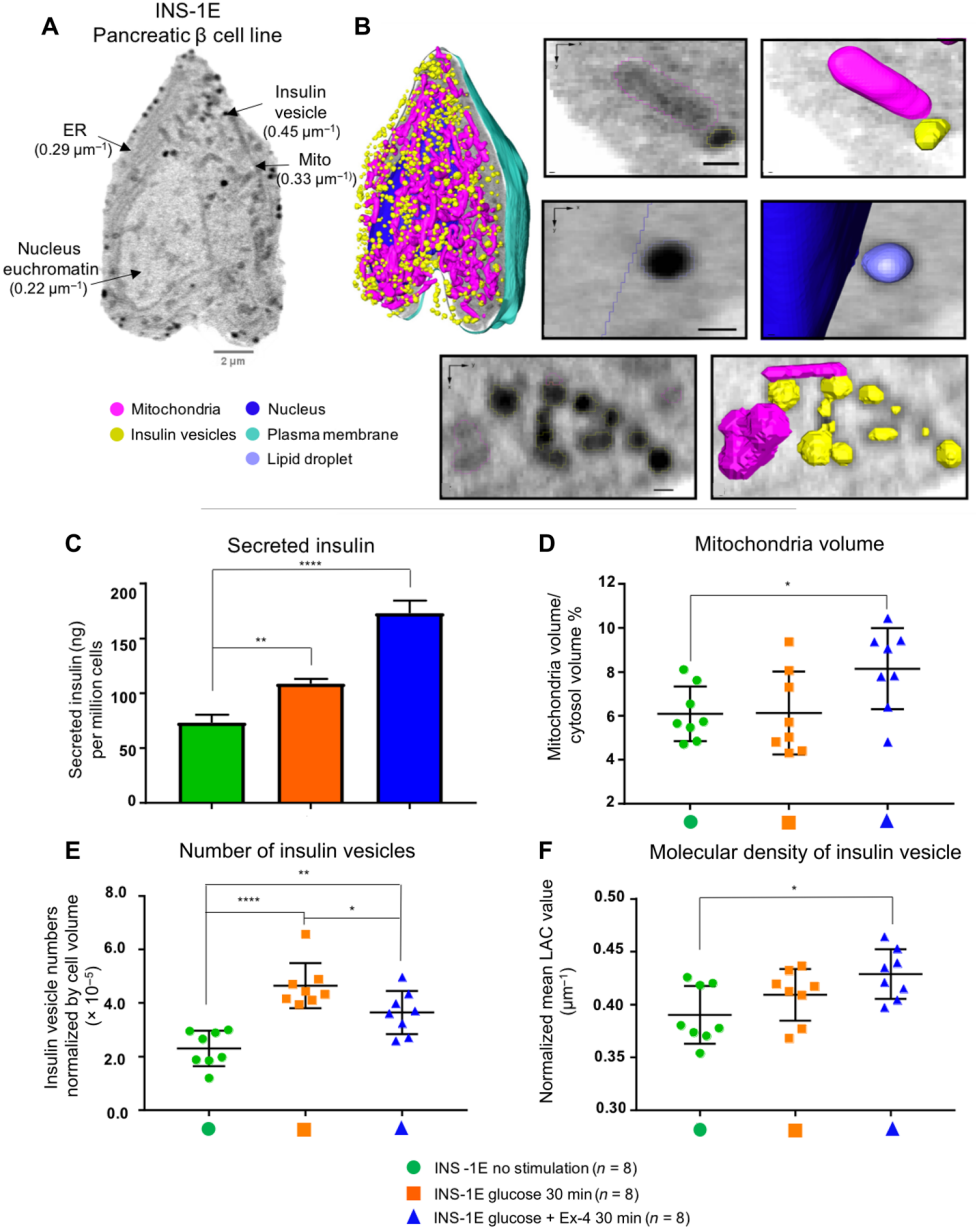
Representative 3D reconstruction of INS-1E cells and the effect of stimulation. (**A**) Representative orthoslice through the 3D reconstruction of a cell, where indicated LAC values are from a representative voxel and (**B**) 3D volumetric images of segmentation. To illustrate the segmentation process, (B) depicts before (left) and after (right) organelle segmentation (scale bars, 350 nm). (**C**) Comparison of insulin secretion with cells in suspension measured in triplicate using enzyme-link immunosorbent assay (ELISA; Mercodia). Statistical differences are shown, where **P = 0.0049 and ***P < 0.0001 (Tukey’s multiple comparison test). (**D**) Plot of mitochondria/cytosol volume ratios, where *P = 0.042 (Dunnett’s multiple comparison test). (**E**) Number of insulin vesicles normalized by cell volume and statistical differences, where ***P < 0.0001, **P = 0.0062, and *P = 0.0436 (Tukey’s multiple comparison test). (**F**) Mean insulin vesicle LAC value for each condition with a difference between unstimulated and glucose + Ex-4 conditions (*P = 0.0106; Dunnett’s multiple comparison test). Error bars in all panels represent SDs. *n* values in figure correspond to (D) to (F).

We used SXT to image INS-1E rat insulinoma cells, which are known to have a glucose dose response similar to rat islets, suggesting that their insulin secretion pathways are comparable (*14*, *15*). The imaging was undertaken for four experimental conditions: no stimulation, stimulation with high glucose (25 mM), stimulation with high glucose in combination with 10 nM of the drug exendin-4 [Ex-4; a glucagon-like peptide-1 receptor (GLP-1R) agonist that potentiates glucose-stimulated insulin secretion], and stimulation with high KCl (50 mM). SXT provides a unique opportunity to use molecular densities (LAC values) to identify cellular structures in the same cell under near-native conditions (Fig. 1A; fig. S3, A to C; and table S1). For individual cells in each experimental condition, we used a combination of morphology and LAC values to segment the PM, nucleus, mitochondria, lipid droplets, and insulin vesicles (Fig. 1B; Materials and Methods). We validated the criteria for insulin vesicle segmentation using three negative controls, including cell lines that do not express insulin (Materials and Methods). This paradigm allowed us to investigate changes in the number, size, and distribution of insulin vesicles and other organelles caused by increased secretory demand after glucose-induced stimulation. We focused on two poststimulation time points that represent the different phases of insulin secretion: the 5-min time point captured the first phase of insulin secretion, and the 30-min time point captured the more prolonged second phase of insulin secretion (*16*).

### Impact of stimuli on general cellular topology

We first focused on the second and more prolonged phase of insulin secretion by investigating cellular structure after 30 min of glucose stimulation. To ensure that the stimulation treatments had the expected effects on insulin secretion, after 30 min of stimulation, the buffer was extracted to measure the levels of secreted insulin using an enzyme-linked immunosorbent assay (ELISA). As expected, we found that both the glucose-stimulated and glucose + Ex-4–stimulated cells secreted more insulin than unstimulated cells. In addition, cells stimulated with glucose alone secreted less insulin than cells costimulated with glucose + Ex-4 (Fig. 1C and table S2). After stimulation, we prepared cells for SXT imaging and organelle segmentation (see Materials and Methods). An orthoslice from the tomogram of each cell used in this study is shown in fig. S2, and the volumes of cells, numbers of insulin vesicles and lipids, and mean LAC values are reported in table S1.

We found no differences in the total volumes of the cell, nucleus, or cytosol between the three conditions (fig. S3, D to F). As a comparison to SXT, we also used fluorescence microscopy to determine that the treatment conditions did not affect the total number of lipid droplets (fig. S3G). There was a significant increase in mitochondria volume upon costimulation with glucose + Ex-4 relative to unstimulated cells (Fig. 1D and table S2). To most accurately compare insulin vesicle numbers across multiple cells, we normalized vesicle numbers by cell volume. We found fewer insulin vesicles in the unstimulated INS-1E cells (319 ± 45) as compared to INS-1E cells stimulated with glucose and glucose + Ex-4 (788 ± 191 and 515 ± 155, respectively) (table S2). A significantly higher number of vesicles were found in cells stimulated with glucose compared to cells costimulated with glucose + Ex-4 (Fig. 1E and table S2). In addition, there was a tendency for smaller vesicle sizes upon stimulation, but this difference was not statistically significant (fig. S3H).

To further characterize the effects of the stimulus conditions on cellular topology, we investigated the molecular densities (LAC values) of multiple organelles. There were no differences in LAC values between unstimulated cells and the 30-min stimulus conditions for the nucleus, cytosol, mitochondria, or lipid droplets (table S1). However, there was a significant increase in the mean LAC value of insulin vesicles in glucose + Ex-4 costimulated cells (0.43 ± 0.02 μm^−1^) relative to unstimulated cells (0.39 ± 0.02 μm^−1^) (Fig. 1F).

### 3D spatial organization of insulin vesicles

**Fig. 2:**
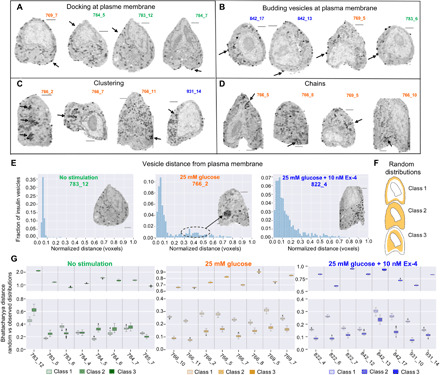
Organization and distributions of insulin vesicles by phenotype within multiple cellular neighborhoods. Four major phenotypes of insulin vesicle organization were observed: (**A**) docking and (**B**) budding outward from the PM, (**C**) clustered, and (**D**) in chains in the cell interior. Four orthoslices are shown for each phenotype from representative cells. (**E**) Distance distribution plots of a representative cell from each stimulation condition. Insulin vesicles dock along the PM in unstimulated INS-1E cells (left) and in large clusters, causing a unique shape to the distribution pattern in those treated with glucose (center). The second smaller peak here (highlighted by the dotted line) corresponds to four insulin vesicle clusters within this cell. In contrast, cells treated with glucose + Ex-4 tended toward a diffuse insulin vesicle distribution (right). Scale bars, 2 μm. (**F**) Three classes of random distribution. Yellow regions represent the allowed volume of random vesicle placement. (**G**) Bhattacharyya distance plots comparing random versus observed distribution of insulin vesicles. For each cell, the observed distribution of insulin vesicles was compared with 50 randomly distributed points shown here as scatter plots. Cell nomenclature refers to capillary number (first number) and cell position within that capillary (number after hyphen).

We investigated the spatial distribution of insulin vesicles, including their locations relative to each other and to other cellular substructures. Before segmentation, four classes of insulin vesicle organization within the cell were immediately identifiable: (i) docked at the PM—a prominent feature of unstimulated INS-1E cells (Fig. 2A) that has also been observed by transmission EM and biochemical methods (*11*, *17*, *18*); (ii) budding from the PM—observed in all conditions, but more noticeable in cells with higher average insulin vesicle diameters (Fig. 2B); (iii) clustered in the interior of the cell—occurred prominently in cells stimulated with glucose alone (Fig. 2C); and (iv) elongated chains—vesicles in a state of active transport that were observed mostly in cells stimulated with glucose alone (Fig. 2D) (*19*). The functions of elongated chains and interior vesicle clusters may be similar but appear visually distinct, and the lack of chains in glucose + Ex-4 cells may be a result of altered trafficking timing rather than different mechanisms of trafficking. These classes appeared to be caused directly by the stimulus condition and the natural functions of the β cell.

We calculated the shortest distance between each insulin vesicle and the PM (Fig. 2E) and found that cells stimulated with glucose ± Ex-4 showed a more uniform vesicle distribution across the cell compared to unstimulated cells. In addition, for several of the stimulated cells, a bimodal spatial distribution was observed. The first peak was associated with the insulin vesicles docked at the PM. The much smaller second peak farther away from the PM corresponds to vesicle clusters in the interior of the cell. For example, in unstimulated cells, most insulin vesicles were within 350 to 525 nm of the PM, while in stimulated cells, the distribution peak is wider, meaning that vesicles were distributed across a broader area extending 700 to 1050 nm away from the PM (Fig. 2E).

We expect that differences in insulin vesicle distributions reflect architectural rearrangements in response to stimulation and are thus likely not random. To test this hypothesis, we measured the similarity between the observed insulin vesicle distributions and a random distribution of points inside the cell by calculating their Bhattacharyya distance (*20*). A low Bhattacharyya distance indicates that a tested distribution is similar to a random distribution. We generated three random distributions, where particles were randomly placed only in (i) a volume close to the PM, (ii) the entire cytoplasmic region, and (iii) a volume that excluded regions close to PM (Fig. 2F and Materials and Methods).

The insulin vesicle distribution in unstimulated cells was most similar to the first class of random distributions and least similar to the third class (Fig. 2G, left panel) because these insulin vesicles were mostly found docked at the PM. There were two exceptions to this observation among all eight cells under the unstimulated condition (cells 783_6 and 785_7), which unusually had more insulin vesicles in the interior of the cell (fig. S2). In contrast, insulin vesicle distributions for glucose and glucose + Ex-4–stimulated cells were more similar to the second class of random distributions, which is similar to a uniform vesicle distribution across the entire cytoplasm (Fig. 2G, middle and right).

It was not immediately clear whether the distance of insulin vesicles from the PM was affected by the deformation in the cell shape due to sample preparation. This is an important consideration for future modeling efforts that might use these data. To investigate, we compared the random distributions of points in the observed irregular cell shapes with the random distribution in a spherical cell of the same volume. A similar trend in the distribution of random points in both the observed and spherical cell shapes would suggest that potential deformation in cell shape caused by sample preparation does not affect the distribution of vesicles from the PM. Figure S4 shows three probability distributions for three cells (one representative cell from each condition). The three distributions are (i) the observed distribution in the observed cell and nucleus shape, (ii) a random distribution in the observed cell and nucleus shape, and (iii) a random distribution in a spherical cell and nucleus shape. A clear difference was detected between observed and random distributions, whereas the random distributions in observed and spherical cell shape were similar. Therefore, we concluded that the observed distributions were not an artifact of cell deformation during sample preparation. However, the position of the nucleus within the cell can influence distributions because of altered space availability for the insulin vesicles.

### Dynamics of cell topology rearrangements during insulin secretion

**Fig. 3:**
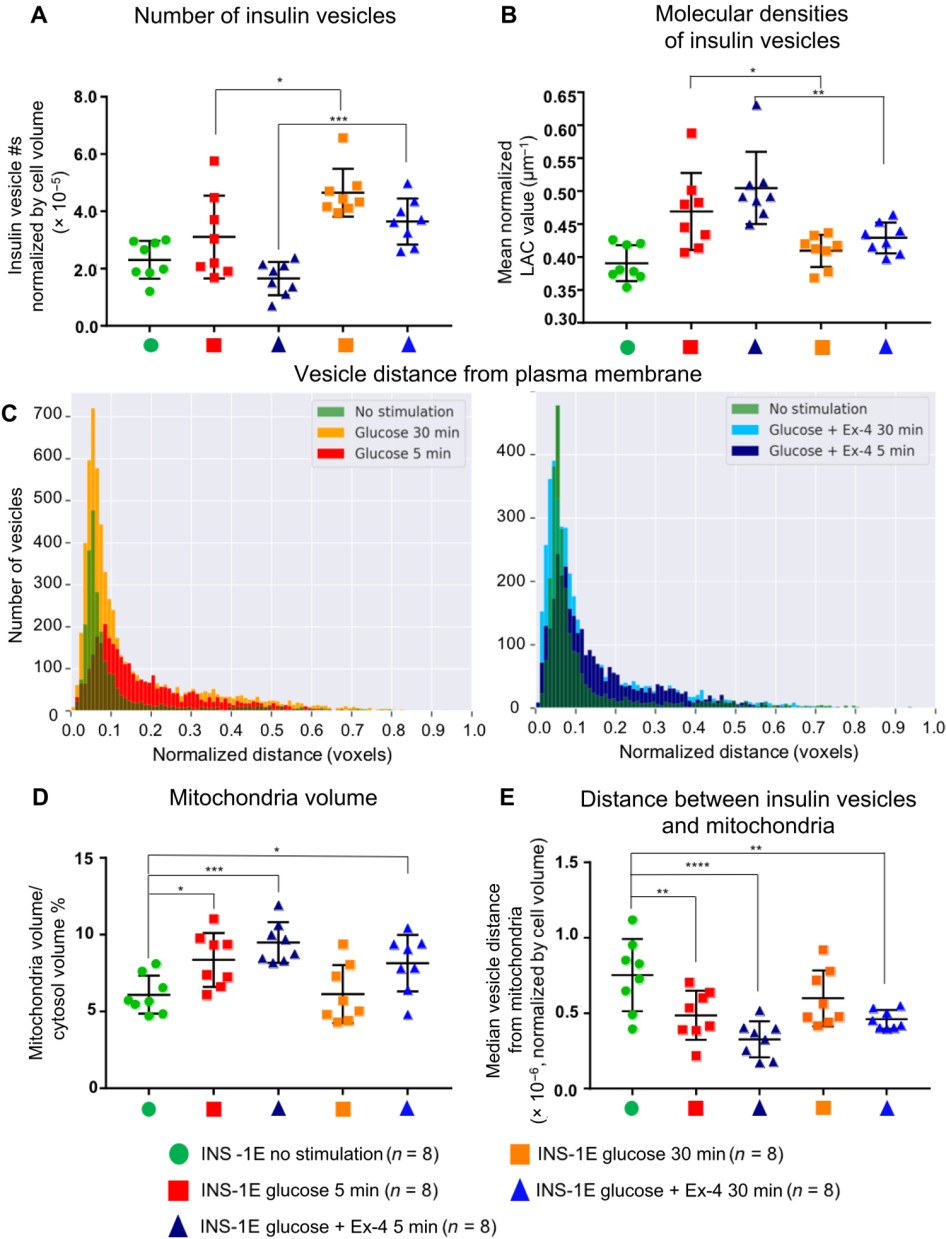
Dynamics of cell topology rearrangements during insulin secretion. (**A**) The number of insulin vesicles normalized by cell volume showing statistical differences between conditions (*P = 0.0118 and **P = 0.0007; Sidak’s multiple comparison test). (**B**) Mean molecular density or LAC value of insulin vesicles in each condition showing statistical differences (*P = 0.0240 and **P = 0.0029; Sidak’s multiple comparison test). (**C**) Plots of the number of insulin vesicles distributed from the PM at both 5- and 30-min time points. (**D**) Plot of the mitochondrial/cytosol volume ratio. Relative to the unstimulated condition, mitochondrial volume was larger for glucose-stimulated cells at 5 min (*P = 0.0252), and the glucose + Ex-4 at 5 and 30 min condition (***P = 0.0007 and *P = 0.0325) (Holm-Sidak’s multiple comparison test). (**E**) Mean mitochondria-insulin vesicle distance for each condition. Relative to the unstimulated condition, there is a smaller distance between insulin vesicles and mitochondria in the glucose-stimulated 5- and 30-min time points (*P = 0.0099 and ****P = 0.0001) and the glucose + Ex-4–stimulated 30-min time point (**P = 0.0040; Dunnett’s multiple comparison test).

After observing substantial structural rearrangements in cells stimulated with glucose ± Ex-4 for 30 min, we investigated the evolution over time of these rearrangements by comparing cell topologies after stimulation with glucose ± Ex-4 for 5 min (Fig. 3 and fig. S5). We observed a significantly higher number of insulin vesicles after 30 min of glucose stimulation relative to their corresponding 5-min time point (Fig. 3A). We included a control to investigate insulin vesicle numbers after stimulation with 50 mM KCl for 5 and 30 min (fig. S5, A to F, and table S1). KCl is often used to mimic the membrane depolarization that occurs during glucose-stimulated insulin secretion; however, KCl exposure is distinct from glucose stimulation because its molecular mechanism relies only on Ca^2+^ influx, while glucose stimulation also induces metabolic changes (*21*). Cells stimulated with KCl for 5 and 30 min have slightly reduced insulin vesicle numbers compared to unstimulated cells (fig. S5B), consistent with the fact that KCl drives secretion but not insulin vesicle biogenesis.

Insulin vesicles showed significantly higher molecular densities (LAC values) in cells stimulated with glucose for 5 min (0.47 ± 0.05 μm^−1^) than for 30 min (0.41 ± 0.02 μm^−1^) (Fig. 3B). Similarly, the mean LAC value of insulin vesicles in cell stimulated with glucose + Ex-4 differed significantly between the 5-min time point (0.51 ± 0.05 μm^−1^) and the 30-min time point (0.43 ± 0.02 μm^−1^). For both stimulations, cells stimulated for 5 min had higher mean insulin vesicle LAC values than unstimulated cells (0.39 ± 0.02 μm^−1^). In contrast, the average insulin vesicle LAC values in cells stimulated with KCl for 5 and 30 min (0.39 ± 0.02 and 0.39 ± 0.01 μm^−1^, respectively) were similar to unstimulated cells (0.39 ± 0.02 μm^−1^) (fig. S5C). Apart from insulin vesicles, we also observed a significant difference in cytoplasmic and nucleic LAC values between unstimulated and cells stimulated with glucose + Ex-4 for 5 min (fig. S5, G to J, and table S1), indicating a rapid and transient alteration of the general cellular state.

Insulin vesicles showed similar distribution patterns between the 5- and 30-min time points, except that the earlier time point had fewer insulin vesicles docked at the PM due to recent secretion without mobilization of new vesicles that is seen at the 30-min time point (Fig. 3C and fig. S6, A and B). We also compared the observed distributions of insulin vesicles after 5 min of glucose ± Ex-4 stimulation with a random distribution of points in the cell as described above. For cells at the 5-min time point, the insulin vesicle distributions were most similar to the second class of random distributions, where vesicles were distributed throughout the cytoplasmic space rather than predominantly closer to or farther away from the PM (fig. S6C). Furthermore, the insulin vesicle distribution patterns for KCl-treated cells appeared similar between 5 and 30 min because we did not detect new vesicles in the interior of the cells (fig. S5D).

The average mitochondrial volume was smaller for unstimulated cells than for cells stimulated with glucose for 5 min, and cells stimulated with glucose + Ex-4 for 5 and 30 min (Fig. 3D). We also analyzed the positions of insulin vesicles relative to the nearest mitochondria in a cell. The median vesicle-mitochondria distance was higher in unstimulated cells compared to those stimulated with glucose for 5 min and glucose + Ex-4 for 5 and 30 min (Fig. 3E). The increase in mitochondria volume in stimulated cells may contribute to the observed differences in mitochondria-vesicle distances compared to unstimulated cells. Thus, we investigated the distribution of the two organelles more closely. We report the fraction of vesicles distributed around the mitochondria within increasing distance increments of 175 nm (fig. S7). Within 175 nm from the mitochondria, there was a higher proportion of vesicles in both the stimulation groups at 5 min and the glucose + Ex-4 at 30 min compared to unstimulated and glucose-stimulated conditions at 30 min. Furthermore, at distances greater than 351 nm surrounding the mitochondria, the unstimulated cells and cells stimulated with glucose for 30 min had a higher proportion of vesicles compared to the other conditions. Therefore, the effect of glucose on the mitochondria-vesicle distance decreased during the second phase of insulin secretion as observed at the 30-min time point, while this effect was prolonged by the presence of Ex-4 (fig. S7). In contrast, there was an increase in mitochondrial volume for cells treated with KCl for 5 min, but no change in the mitochondria-vesicle distances compared to unstimulated cells (fig. S6, E and F).

**Fig. 4:**
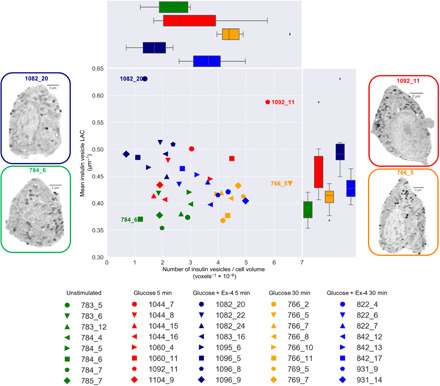
Condition clustering and cell-to-cell heterogeneity in topology. Mean insulin vesicle LAC value versus numbers of insulin vesicles normalized by cell volume for each cell. Each point represents one cell as indicated in the key. Representative orthoslices from the 3D reconstructions for the cells at the extremes of the plots are shown for reference. Diamonds in the box and whisker plots represent points outside the SD.

To better characterize the effects of each treatment on cellular topology, we plotted the average insulin vesicle LAC value versus vesicle concentration for each cell. The different conditions cluster into distinct groups (Fig. 4). This analysis highlights cell-to-cell variability within each condition and illustrates the phenotypic response across the examined treatment conditions.

## DISCUSSION

We demonstrate that our SXT topology mapping pipeline can capture the cellular topology changes induced by both drug treatments and different stimulus time points. Our results are consistent with many previous studies on insulin vesicle diameter, observation of insulin vesicles docked to the PM (*3*, *11*, *17*), and close association of insulin vesicles and mitochondria (*22*). We detect fewer insulin vesicles than have been reported by other studies of primary cells (*3*, *23*). It is known that INS-1E cells have a lower insulin content (*15*), but a recent report using 3D structured illumination microscopy on fixed cells with an indirect immunolabeling strategy (*24*) found more insulin vesicles in unstimulated INS-1E cells than we do with SXT. With the indirect immunolabeling approach, it is difficult to identify distinct or individual insulin vesicles and, therefore, this technique could overestimate the total numbers. In addition, it is possible that in using SXT, we do not identify all immature vesicles in the interior of the cell or some vesicles fused to the PM. In these cases, the lack of crystalline insulin would reduce the molecular density of individual vesicles, making these vesicles more difficult to detect with SXT. Future efforts combining correlative fluorescence microscopy with SXT (*1*) will be useful for monitoring both immature and mature vesicles in the context of all organelle structures.

Within 5 min of glucose stimulation, we observed an increase in the average molecular density of insulin vesicles, but not an increase in vesicle numbers relative to unstimulated cells. After 30 min of glucose stimulation, there is an increased number of vesicles relative to unstimulated cells, which could be caused by a rapid maturation or acidification and subsequent crystallization of insulin within immature vesicles (*11*, *25*), and/or the biosynthesis of new vesicles. We did not observe changes in insulin vesicle molecular densities or increased numbers of insulin vesicles in the KCl-stimulated cells, suggesting that the combined effect of calcium influx and glucose metabolism are responsible for these effects. Costimulation with Ex-4 prolongs the effects on insulin vesicle molecular densities, and we found fewer insulin vesicles in cells from the glucose + Ex-4 condition than in those from the glucose alone condition after 30 min of stimulation (Fig. 3, A and B). These results suggest that Ex-4 is affecting the trafficking system to promote insulin secretion. It does so by altering the insulin vesicle maturation process via rapid acidification, increased packing of insulin within each vesicle, and/or altering the recycling of old vesicles that would normally be degraded. Molecules that affect insulin packing and promote the efficient use of all insulin vesicles have the potential to improve insulin output without causing additional stress to β cells (*26*). An in-depth spatial proteomics analysis corresponding to both conditions and time points will help to elucidate the effect of this increased molecular density (*27*).

We could gain further insights into the vesicle trafficking pathway by analyzing the physical positions of insulin vesicles and mitochondria in relation to the ER and Golgi. However, currently, we are limited in our ability to segment these organelles, both because there are challenges in distinguishing them from other organelles and because of the time-consuming nature of the semi-automatic segmentation process that limits the number of cells that can be analyzed. Future studies could leverage correlative fluorescence microscopy and SXT (*1*) of the same cell by labeling live cells with ER and Golgi markers, which will enhance our ability to distinguish these features for segmentation and allow automated machine-learning segmentation approaches (*28*). These approaches will be useful as many questions remain on the process of vesicle maturation (*11*, *29*).

We expected differences in insulin vesicle distributions after glucose stimulation, but did not anticipate the rapid (i.e., within 5 min) and significant increase in mitochondrial volume. Others have reported a similar increase in mitochondrial volumes with GLP-1R stimulation (*30*), but the mechanism for this change remains unclear and may be different depending on the stimulation. This rapid increase in mitochondrial volume was accompanied by a trend of increased molecular densities inside mitochondria, indicating a biochemical change such as adenosine triphosphate (ATP) production corresponding to glucose metabolism (*31*). We also observed a closer proximity of insulin vesicles to mitochondria in the cells stimulated with glucose + Ex-4 (Fig. 3E). Others have hypothesized that this close association may facilitate metabolism-secretion coupling (*32*), as ATP enables the movement of insulin vesicles for exocytosis (*33*–*35*).

Future efforts will involve mapping human primary β cells to investigate how disease alters subcellular structure and how pharmacological interventions might rescue insulin deficiencies. Furthermore, this approach can be applied to other secretory cells, such as α cells, and can also be used to investigate subcellular changes that occur during stem cell differentiation. Because SXT allows high-throughput quantification of intact near-native state cells, more conditions and time points can be screened for follow-up studies in islets. Recent availability of large-scale EM datasets of islets (*36*, *37*) provides an opportunity for mapping cell heterogeneities in subcellular structure to specific positions within the islet (interior versus exterior or nearby vasculature). Subcellular quantification of these 3D datasets is still limited because of the lack of whole-cell organelle segmentations. As machine learning–driven autosegmentation continues to improve, there is potential to analyze the 3D reconstructions from these datasets with the analysis pipeline described here to quantify organelle distributions within the cell. Together with the assessment of molecular density changes from SXT data, these approaches have great promise for investigating subcellular structures in health and disease.

**Fig. 5:**
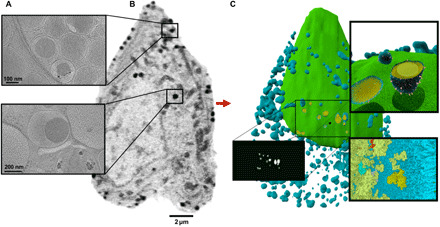
SXT mesoscale maps will be a foundation for future of whole-cell modeling efforts. (**A**) Representative electron tomograms of INS-1E cells depict the different subcellular neighborhood environments in the periphery and center of the cell as indicated. (**B**) Representative SXT orthoslice depicting whole-cell architecture. (**C**) 3D molecular model of an INS-1E cell generated using cellPACK (*38*) from segmented SXT data. Nucleus is shown in green, insulin vesicles are shown in blue, and core of insulin vesicles is shown in yellow. The mesh of each structure is textured with lipids, and the zoom views depict the atomic details of protein packing. The black widow is a rendering of the segmented vesicle mask used to generate the model. Image courtesy of L. Autin and A. Olson.

Many of our results would not have been detected with traditional imaging modalities, underlining the importance of unbiased mapping of subcellular organization at the mesoscale. The resulting 3D maps represent a critical resource for integrating multiscale data for structural cell models (Fig. 5) that describe how all the cellular components fit together and interact in a crowded environment (*38*). To approach the challenge of mapping whole cells, we must integrate multiscale data acquired from multiple imaging strategies. While SXT provides the mesoscale map, cryo–electron tomography provides molecular resolution windows into specific cellular neighborhoods (*39*). Corroborating these two methods, super-resolution microscopy provides dynamic live cell results, and together, these results should be integrated into a comprehensive spatial model. We expect that our SXT pipeline will contribute important data toward assembling an integrative model of an entire β cell (*13*). Such a model and its underlying datasets will provide a powerful avenue to guide efforts on probing the effects of drugs on insulin secretion.

## MATERIALS AND METHODS

### Experimental design

#### Experimental model and subject details

Human embryonic kidney (HEK) 293 cells (American Type Culture Collection, CRL-11268) were cultured in Dulbecco’s Modified Eagle’s medium high glucose (DMEM-HG), 10% fetal bovine serum (FBS), and 1× penicillin-streptomycin and seeded in media at a density of 2.5 × 10^4^ cells/cm^2^. 1.1B4 cells (Sigma-Aldrich, 10012801) were cultured in RPMI media, 10% FBS, 2 mM glutamine, and 1× penicillin-streptomycin and seeded in media at a density of 2.0 × 10^4^ cells/cm^2^. INS-1E cells [Addex Bio, C0018009; RRID (Research Resource Identifiers) accession number: CVCL_0351] were cultured in T75 flasks with Addex Bio Optimized RPMI media supplemented with 10% FBS, 1× penicillin-streptomycin, and 0.05 mM β-mercaptoethanol and seeded in media at a density of 8.0 × 10^4^ cells/cm^2^. All cells were grown in either T25 or T75 flasks in 37°C incubators with 5% CO_2_. We used cells ranging from 45 to 55 passages. Before purchasing, all cell lines were authenticated by each supplier. INS-1E cells were analyzed using COI Assay by the supplier (Addex Bio) to ensure no interspecies contamination (*40*, *41*). In addition, we performed RNA sequencing to confirm the presence of insulin, observed the cells under microscopy to identify changes in morphology and attachment properties, and maintained growth curves to monitor cell doubling time, growth trends, and passage number. We monitor glucose-induced insulin secretion weekly to track the health of the cells over time. The most ideal source for INS-1E cells for future work is directly from the Pierre Maechler laboratory (University of Geneva), who originally developed the INS-1E cell line. However, we observe similar insulin secretion responses and morphology between INS-1E cells acquired from Addex Bio and the Maechler laboratory. There is an average of 2.51 ± 0.09 μg/million cells in the Addex Bio cells, which is comparable to the 2.30 ± 0.11 μg/million cells reported in the founder cell line (*15*). In addition, we observe a 6.7-fold increase in insulin secretion with glucose at 30 min, consistent with the 6-fold change observed in the founder paper (*15*).

#### Measurement of secreted insulin

Insulin assays were performed similarly to previous detailed reports (*15*, *42*). Forty-eight hours before assay, cells were plated in a 24-well plate with 2-cm^2^ surface area in 1 ml of standard media and incubated at 37°C in 5% CO_2_. The assay was performed at 60 to 80% confluency, and before glucose treatment, the cells were incubated in Krebs-Ringer bicarbonate buffer (KREBs) (115 mM NaCl, 24 mM NaHCO_3_, 5 mM KCl, 1 mM MgCl_2_, and 1 mM CaCl_2_) supplemented with 10 mM Hepes (pH 7.4), 0.2% bovine serum albumin, and 1.1 mM glucose for 30 min at 37°C. The glucose starvation buffer was then removed via pipette and replaced with 500 μl of buffer containing either no glucose, 25 mM glucose, or 25 mM glucose + 10 nM Ex-4 and incubated for 30 min at 37°C. The supernatant was removed from cells and spun at 4°C for 10 min in a microcentrifuge to ensure that no detached cells were analyzed in the measurement. Secreted insulin was measured and analyzed according to kit instructions (Mercodia). This method was used for quickly investigating time courses and different drug treatments to generate the secreted insulin data in fig. S5A.

#### Glucose-stimulated insulin secretion with INS-1E cells before SXT

Glucose stimulation for samples prepared for SXT was done as described above except samples were treated in solution in Eppendorf tubes after cells were removed from the T75 flasks. This allowed a gentle stimulation before inserting cells in glass capillaries. Insulin secretion was then measured as described above except the cells were in suspension instead of plated on tissues culture wells. We observed similar trends and magnitudes for both methods of inducing insulin secretion (Fig. 1C).

#### Sample preparation for SXT

Cells were stimulated as indicated above. The unstimulated cells were placed on ice immediately following the 30-min starvation step, and either 5 or 30 min after stimulation, cells were placed on ice in Eppendorf tubes in preparation for cryo-fixation in glass capillaries. Cells were loaded into thin-walled (200 nm) glass capillaries (in KREBs buffer as described above) and rapidly frozen by plunging into liquid nitrogen–cooled propane and stored in liquid nitrogen until data collection (*43*).

#### SXT data collection

Data were collected as previously described (*43*, *44*). Briefly, projection images were collected at 517 eV using XM-2, the National Center for X-ray Tomography soft x-ray microscope at the Advanced Light Source of Lawrence Berkeley National Laboratory; the microscope was equipped with a 60-nm resolution defining objective lens. During data collection, the cells were maintained in a stream of helium gas cooled to liquid nitrogen temperatures (*45*, *46*), which allows collection of projection images while reducing the effects of exposure to radiation. Projection images were collected sequentially around a rotation axis of 180° in 2° increments. Depending on the synchrotron ring current, an exposure time of 140 to 300 ms was used. Tomographic reconstructions were calculated using the iterative reconstruction method as previously described (*47*–*49*). LAC values were determined as previously described (*50*). Each voxel represents 35 nm^3^. Representative cells were selected for each experimental condition (fig. S2) based on quality of the tomograms and of the health of the cell (i.e., intact cell). For negative controls, four cells were chosen with the same criteria (fig. S2).

#### LAC and segmentations

As mentioned in the main text, the absorption of x-rays by the specimen adheres to the Beer-Lambert law and, therefore, is linear, quantitative, and a function of thickness and chemical composition (*51*). As previously described, highly solvated regions of the cell appeared relatively transparent to x-rays compared with regions that were densely packed with carbon or biomolecules. After cells were reconstructed, they were segmented by bounding regions of similar x-ray attenuation characteristics, or molecular densities. The molecular densities were quantified for each voxel in the reconstruction, which is known as the LAC.

Segmentation of the nucleus and PM was undertaken with Amira 2019.1 (FEI) using the “paintbrush” tool, where the outer edge of the nucleus was manually traced orthoslice by orthoslice, and using the “interpolation” feature. Tracing accuracy was confirmed in three dimensions. Segmentation of the lipid droplets, insulin vesicles, and mitochondria was done using the semi-automatic “magic wand” tool, where voxels of specific LAC values could be selected for segmentation (*43*).

The following criteria were used for segmentation for all cell types and treatment conditions used. The nucleus was easily identified by its large size and distinct nuclear membrane with noticeable euchromatin, heterochromatin, and nucleoli phases. For simplicity, we segmented the entire nucleus as a whole and observed an average nucleus LAC value of 0.26 ± 0.02 μm^−1^ (Fig. 1A and table S1, unstimulated cells). The mitochondria were identified by their characteristic elongated morphology and size with an average LAC value of 0.34 ± 0.02 μm^−1^ (Fig. 1A and table S1, unstimulated cells). The ER had classical thin layering of membranes as expected (Fig. 1A), and because of the thin morphology of this organelle, we did not segment the ER in this study. Lipid droplets were identified by their distinctly high LAC values corresponding to dense lipids (*1*), and spherical structures containing a maximum LAC value of ≥0.68 μm^−1^ were labeled as lipid droplets. The average LAC value for all voxels in the lipid droplets was 0.59 ± 0.01 μm^−1^ (table S1, unstimulated cells). Insulin secretory vesicles were particularly easy to identify using SXT, because insulin is packed tightly in vesicles, often forming protein crystals in situ (*52*). There was average insulin vesicle LAC of 0.39 ± 0.02 μm^−1^ (table S1, unstimulated cells). Because insulin vesicles change in density as they mature, a larger range of LAC values (0.35 to 0.67 μm^−1^) was used along with morphology and size to identify insulin vesicles in all conditions. For identifying insulin vesicles, a 90-nm-diameter cutoff was used to exclude small synaptic-like microvesicles that contain γ-aminobutyric acid, which are not a focus of this study (*53*, *54*). While similar in shape to lipid droplets, insulin vesicles are smaller than lipid droplets with an average diameter of 243 ± 39 and 319 ± 45 nm, respectively (table S1), and the differences in LAC values of lipid droplets and insulin vesicles for each cell are easily distinguishable during segmentation (table S1 and depicted in fig. S3, A to C). The strict criteria for distinguishing between lipid droplets and insulin vesicles were primarily used for the negative controls described below and provide clear protocol for multiple people to segment datasets. We found that one person could segment eight cells in a matter of 2 days, and quality control was performed by a second individual, which required an additional day.

There was no difference in the average glass capillary LAC values between different conditions (fig. S5G); however, there was small cell-to-cell variability between capillary LAC values in each cell measurement (table S1). We therefore normalized the SXT LAC values of each organelle with respect to the LAC value of the glass capillary for each cell to rescue any minor systematic errors. The raw and normalized data are shown in table S1.

#### Validation of insulin vesicle segmentation criteria

We used three negative control conditions of cells that do not have insulin vesicles: (i) the 1.1B4 human β cell line that does not express insulin transcripts (*55*), (ii) unstimulated HEK cells, and (iii) glucose-stimulated HEK cells. We identified very few structures that matched our criteria for insulin vesicle segmentation in 1.1B4 (6% of unstimulated cells), HEK (9% of unstimulated cells), and HEK glucose–stimulated cells (5% of INS-1E glucose 30 min cells). All three INS-1E conditions had a significantly higher normalized number of insulin vesicles relative to the control 1.1B4, HEK, and glucose-stimulated HEK cells (fig. S8A and table S1).

#### Lipid droplet fluorescence microscopy

INS-1E cells for fluorescence imaging were treated similar to ones used for insulin secretion assay, as mentioned above. INS-1E cells were plated at 60,000 to 70,000 cm^−2^ on an eight-well glass-bottom plate with a surface area of 1.5 cm^2^ in 200 μl of standard media each well, 72 hours before imaging. Before imaging, cells were starved in glucose starvation KREBs buffer for 30 min. The glucose starvation buffer was then replaced with 200 μl of treatment KREBs buffer with no glucose, with 25 mM glucose, or with 25 mM glucose + 10 nM Ex-4 and incubated for 25 min at 37°C, 5% CO_2_. The treatment buffer was then replaced with the same treatment buffer containing 2 μM BODIPY and incubated for 5 min. After incubation, the dyed cells were washed three times with treatment buffer and imaged live in treatment buffer and at 37°C with 5% CO_2_.

Cells were imaged in a Carl Zeiss 780 inverted confocal microscope with a 40× water immersion objective. A monochrome laser at 488 nm was used for excitation, and the emission spectrum was collected at 500 to 550 nm. Images were captured at 1048 pixel × 1048 pixel resolution with optimal field of view and with Z-stacks across the entire thickness of the cell monolayer at 0.56 μm per slice. Image analysis was performed in Imaris (ver. 3.12; Oxford Instruments) using the cell generation pipeline. All lipid droplets in an image were counted and divided by the number of cells in an image to yield the result. The imaging experiment was done within 15 min after the pigmentation process to avoid nonspecific tagging.

### Statistical analysis

All statistical analyses were undertaken using Prism (version 7.0 for Windows, GraphPad Software, La Jolla, CA) unless otherwise stated. All analysis of variance (ANOVA) analyses were performed to determine whether two or more means are equal between conditions, and the follow-up post hoc analysis was done to determine the specific condition means that differed from each other. Full statistical reports for ANOVA and corresponding post hoc analyses are in table S2. Descriptions of post hoc tests used are included below.

#### Insulin secretion

The mean amount of secreted insulin for conditions was plotted as a bar graph, with error bars representing SD (*n* = 3). A Tukey’s multiple comparison analysis was used to compare mean values of insulin secretion for each condition and determine whether different stimulus conditions had a significant effect on insulin secretion (Fig. 1C).

#### Organelle volumes

The mean volume in voxels was plotted for each cell studied, with error bars representing SD for each condition (*n* = 8; except KCl condition where *n* = 4). A Dunnett’s multiple comparison test was used to compare the mean mitochondrial/cytosol volume ratio of unstimulated cells versus the 30-min stimulation conditions (Fig. 1D) or versus KCl stimulation conditions (fig. S5E). A Holm-Sidak’s multiple comparison test was used to compare the mean mitochondria volume of unstimulated cells compared to glucose ± Ex-4 5 and 30 min conditions (Fig. 3D).

#### Number of insulin vesicles normalized by cell volume

The numbers of insulin vesicles were normalized by cell volume for all cells used in this study. The data are presented as means for each cell represented by one point and with error bars representing SD for each condition (*n* = 8; except for HEK, HEK glucose, 1.1B4, and KCl conditions, where *n* = 4). A Tukey’s multiple comparison analysis was used to compare the means of each condition and determine whether different stimulus conditions had a significant effect on the number of insulin vesicles (Fig. 1E and fig. S8). A Sidak’s multiple comparison test was used to compare the mean number of insulin vesicles for the preselected groups: (i) unstimulated versus glucose 5 min, (ii) unstimulated versus glucose + Ex-4 5 min, (iii) unstimulated versus glucose 30 min, (iv) unstimulated versus glucose + Ex-4 30 min, (v) glucose 5 min versus glucose 30 min, and (vi) glucose + Ex-4 5 min versus glucose + Ex-4 30 min (Fig. 3A).

#### LAC value comparisons

The mean LAC value for specific organelles of each cell was plotted, with error bars representing SD for each condition. Means were obtained for each cell by generating the mean of all voxels labeled as that organelle. A Dunnett’s multiple comparison test was performed to compare the mean insulin vesicle LAC value of the unstimulated condition to glucose 30 min and glucose + Ex-4 30 min (Fig. 1F). A Sidak’s multiple comparison test was performed to compare the mean insulin vesicle LAC value for the following preselected pairs: (i) glucose 5 min versus glucose 30 min, (ii) glucose + Ex-4 5 min versus glucose + Ex-4 30 min, (iii) unstimulated versus glucose 5 min, and (iv) unstimulated versus glucose + Ex-4 5 min (Fig. 3B). A Dunnett’s multiple comparison test was performed to compare the mean cytoplasm, nucleus, and mitochondria LAC value between unstimulated and all other conditions (fig. S5, G to J).

#### Organelle distance calculations

##### Mitochondria-insulin vesicle distances

Each insulin vesicle-mitochondria distance was normalized by the cell volume to consider the difference in cell size and therefore insulin vesicle concentration within the cell. A Dunnett’s multiple comparison test was performed to compare the mean distance between mitochondria and insulin vesicles of unstimulated versus the other conditions (Fig. 3E).

##### Insulin vesicle random distribution in observed cell shape

First, we defined the cytoplasmic volume by excluding the nuclear and mitochondrial volume from the segmented cell in the tomographic image. To generate random insulin vesicle placements in each cell, selected points were randomly placed only in the cytoplasmic volume. For each cell, the same number of vesicles was chosen as observed in the corresponding cell. Selected points were at least four voxels apart to consider their excluded volume. To define class 1 and class 3 random distance distributions, we imposed additional constraints in the point selection procedure. First, a distance threshold was set equal to 20% of the maximum possible distance between a chosen point and the PM. For class 1 distributions, random point positions were only selected if their distance to the PM was smaller than the distance threshold. For class 3 distance distributions, random point positions were only selected if their distance to the PM was larger than the distance threshold.

##### Creating a random distribution in the spherical cell shape

To characterize the effect of cellular and nuclear shape on insulin vesicle distributions, new simulated random distributions were created for cells with spherical shapes and spherical nuclei, using volumes obtained from tomograms. The analysis was performed for three samples: 783_12 with no stimulation, 766_2 with glucose stimulation, and 822_4 with glucose + Ex-4 stimulation. For each cell, 1000 μs of Brownian dynamics simulations was performed with random starting configurations and free diffusion using the Integrative Modeling Platform (IMP) (*56*). Subsequently, the distribution was analyzed every 50 μs for simulations that were at least 900 μs in duration. As can be seen in fig. S4, the fraction of insulin vesicles is, as expected, proportional to the volumes of spherical slices along the distances to PM normalized by cell radius.

##### Probability of vesicle distance from PM

The distance of each vesicle center from the PM was calculated using Euclidean distance transform. Because cells vary in size and shape, we normalized the observed distance in the tomogram, *d*^obs^, between an insulin vesicle and the PM by the maximum distance possible in the respective cell. The normalized distance *d* is defined as *d* = *d*^obs^/*D*_C_, where *D*_C_ is the maximum possible distance from the PM in this particular cell. Therefore, the binned range of normalized distances is [0,1]. Distances were binned to generate the distance distribution histograms. Probability distributions were generated by dividing the value of each histogram bin by the total number of insulin vesicles to give the probability.

##### Bhattacharyya distance

To quantify the dissimilarity between two probability distribution functions, the Bhattacharyya distance was calculated (*20*). We compared the probability distance distribution of insulin vesicles observed experimentally with those generated by randomly placing insulin vesicles into the same cell volume. Specifically, we compared the probability of finding an insulin vesicle at the observed distance, *d*, in cell, *c*, with the probability of finding an insulin vesicle at distance *d* using a random distribution of insulin vesicles. For each cell, we computed random distribution 50 times within each class. Measurements of these distances are shown in Fig. 2G and fig. S6C.

## Supplementary Material

http://advances.sciencemag.org/cgi/content/full/6/50/eabc8262/DC1

Table S1

Adobe PDF - abc8262_SM.pdf

Visualizing subcellular rearrangements in intact β cells using soft x-ray tomography

## SUPPLEMENTARY MATERIALS

Supplementary material for this article is available at http://advances.sciencemag.org/cgi/content/full/6/50/eabc8262/DC1

## Appendix

View/request a protocol for this paper from *Bio-protocol*.
