# Supplementary material for: Visualizing subcellular rearrangements in intact β cells using soft x-ray tomography
Source: Sci Adv. 2020 Dec 9;6(50):eabc8262. doi: 10.1126/sciadv.abc8262 (PMC7725475; doi:10.1126/sciadv.abc8262)
Supplement: http://advances.sciencemag.org/cgi/content/full/6/50/eabc8262/DC1 [file supp_6_50_eabc8262__index.html]

Science Advances | Science AdvancesAAASSearchScience AdvancesMenu

## Supplementary Materials

# Visualizing subcellular rearrangements in intact β cells using soft x-ray tomography

Kate L. White, Jitin Singla, Valentina Loconte, Jian-Hua Chen, Axel Ekman, Liping Sun, Xianjun Zhang, John Paul Francis, Angdi Li, Wen Lin, Kaylee Tseng, Gerry McDermott, Frank Alber, Andrej Sali, Carolyn Larabell, Raymond C. Stevens

Download Supplement

**The PDF file includes:**

- Figs. S1 to S8
- Table S2
- Legend for table S1

**Other Supplementary Material for this manuscript includes the following:**

- Table S1

**Files in this Data Supplement:**

- Adobe PDF - abc8262\_SM.pdf
- abc8262\_Table\_S1.xlsx
