# Supplementary material for: Visualizing subcellular rearrangements in intact β cells using soft x-ray tomography
Source: Sci Adv. 2020 Dec 9;6(50):eabc8262. doi: 10.1126/sciadv.abc8262 (PMC7725475; doi:10.1126/sciadv.abc8262)
Supplement: http://advances.sciencemag.org/cgi/content/full/6/50/eabc8262/DC1 [file supp_6_50_eabc8262__1.pdf]

Science Advances
AAAS

advances.sciencemag.org/cgi/content/full/6/50/eabc8262/DC1

Supplementary Materials for

Visualizing subcellular rearrangements in intact β cells using soft x-ray tomography

Kate L. White*, Jitin Singla, Valentina Loconte, Jian-Hua Chen, Axel Ekman, Liping Sun, Xianjun Zhang, John Paul Francis, Angdi Li, Wen Lin, Kaylee Tseng, Gerry McDermott, Frank Alber, Andrej Sali*, Carolyn Larabell*, Raymond C. Stevens*

*Corresponding author. Email: stevens@usc.edu (R.C.S.); calarabell@lbl.gov (C.L.); sali@salilab.org (A.S.); katewhit@usc.edu (K.L.W.)

Published 9 December 2020, *Sci. Adv.* **6**, eabc8262 (2020)
DOI: 10.1126/sciadv.abc8262

The PDF file includes:

Figs. S1 to S8
Table S2
Legend for table S1

Other Supplementary Material for this manuscript includes the following:

(available at advances.sciencemag.org/cgi/content/full/6/50/eabc8262/DC1)

Table S1

Supplementary Materials

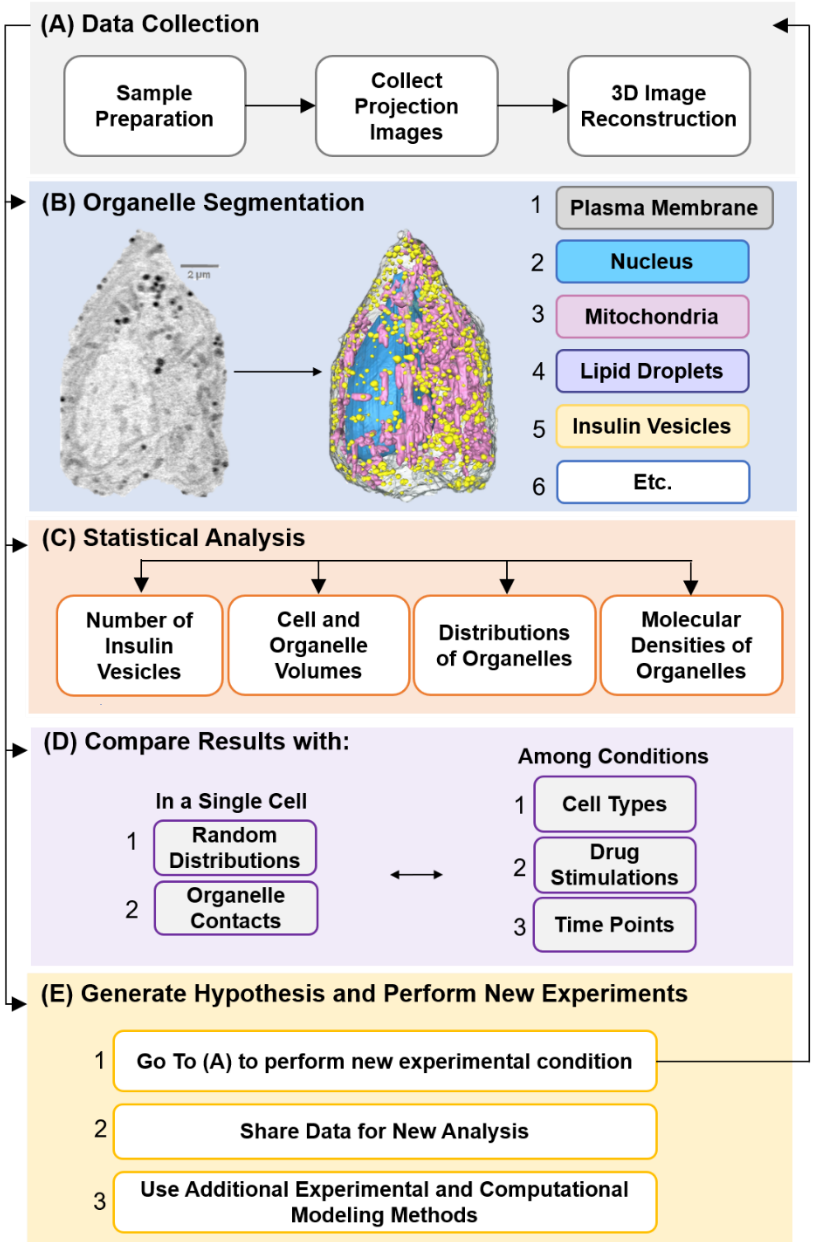

Fig. S1. Pipeline of SXT data collection and analysis.

(**A**) Samples are prepared by fast freezing cells in glass capillaries after stimulation. Projection images are collected for all cells in each condition and the 3D reconstruction of the tomogram is performed automatically. The process of collecting and reconstructing images takes <5 min for each cell. (**B**) Semi-automatic organelle segmentation is performed using Amira software (FEI, ThermoFisher) using a combination of LAC values and structural features. Plasma membrane shown in transparent gray, nucleus in blue, mitochondria in pink, lipid droplets in light blue, insulin

vesicles in yellow. (**C**) Statistical analysis is performed using the segmented organelle masks to determine the number, volume, distribution, and molecular density of organelles. (**D**) These results were compared between cell types and drug stimulations at different timepoints. Random and observed distributions of organelles were compared to determine both how closely specific organelles associate with one another and the topology signatures of specific conditions. (**E**) The final step is to share the results with the scientific community for additional analysis, additional experimental computational methods to investigate new hypotheses generated.

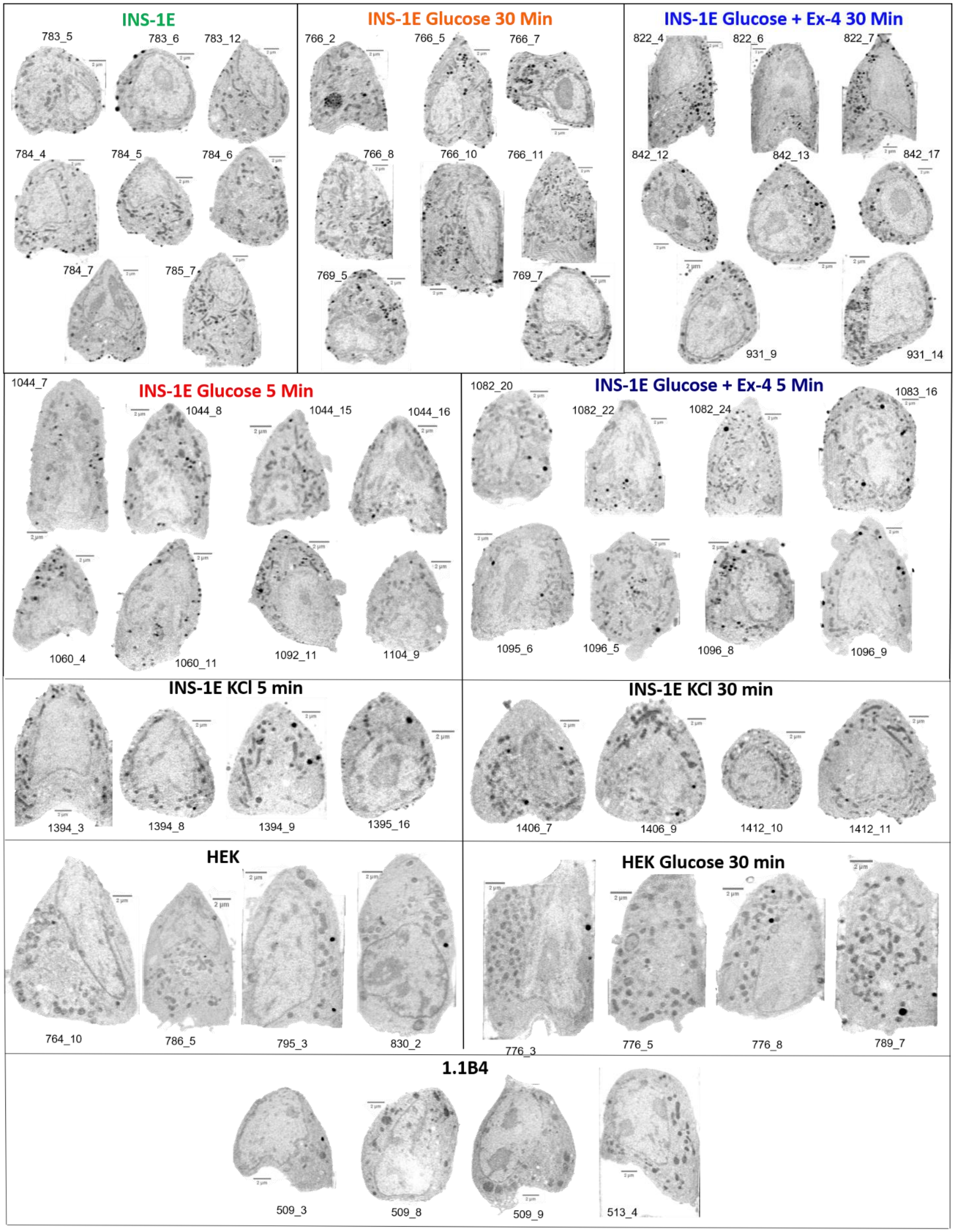

Fig. S2. Orthoslices of all cells used in analysis. Related to Fig. 2. 2D orthoslices with scale bars were generated in Fiji (Image J2). All glass capillaries were removed from the image to

allow for easier visual comparisons. Scale bar in all images is 2 µm. Cell nomenclature refers to capillary number (first number) and cell position within that capillary (number after hyphen).

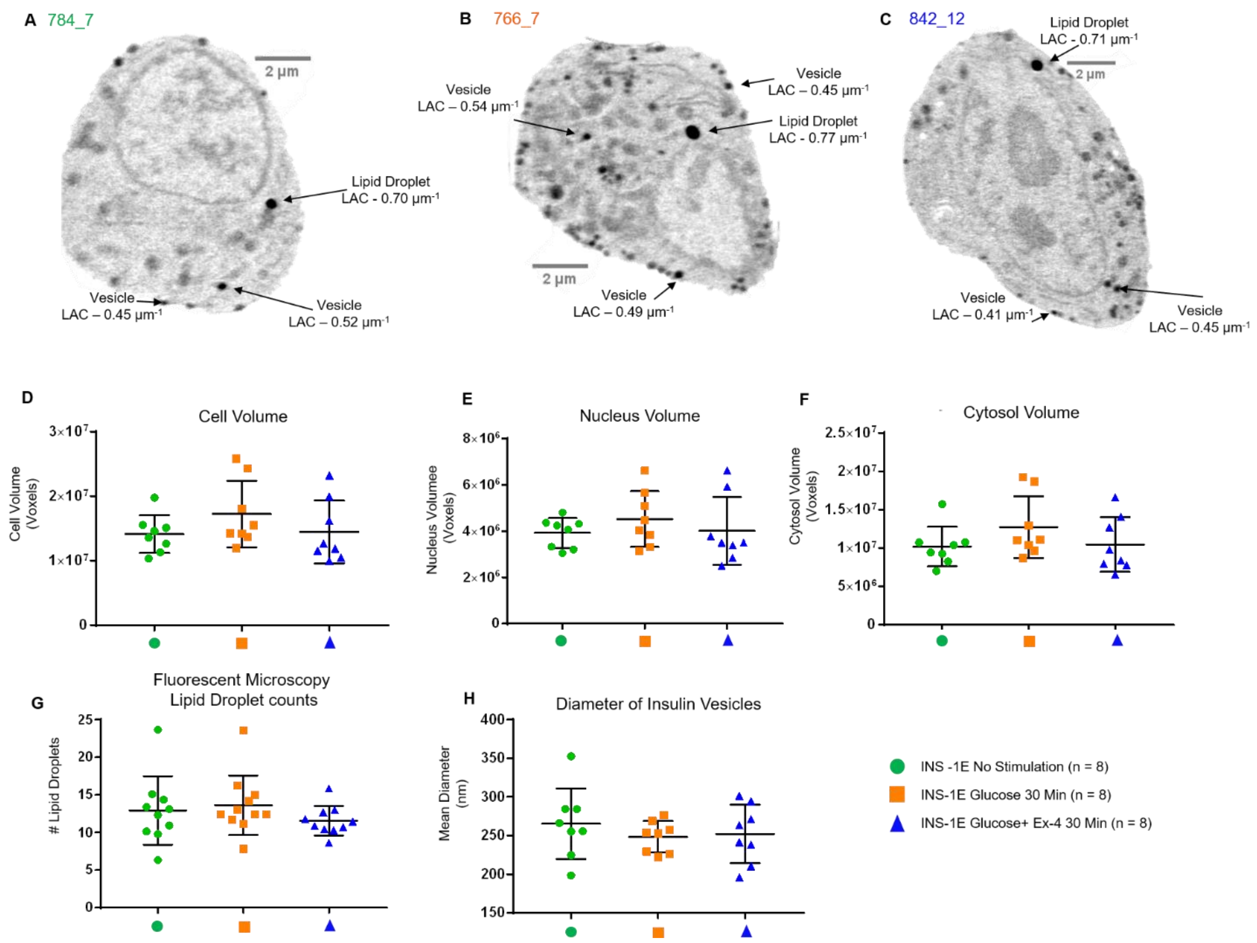

Fig. S3. Comparison of insulin vesicles and lipid droplets, showing volumes of multiple structures. Related to Fig. 2. (**A-C**) Comparison of lipid droplets and insulin vesicles are shown in representative 2D orthoslices from the 3D tomograms for one cell of each condition. Plots of cell (**D**), nucleus (**E**), and cytosol (**F**) volumes, and diameter of insulin vesicles (**G**) are provided. (**H**) Plot of lipid droplet numbers detected by fluorescence microscopy reveal no effect of stimulation conditions. N values refer to panels **D-H.**

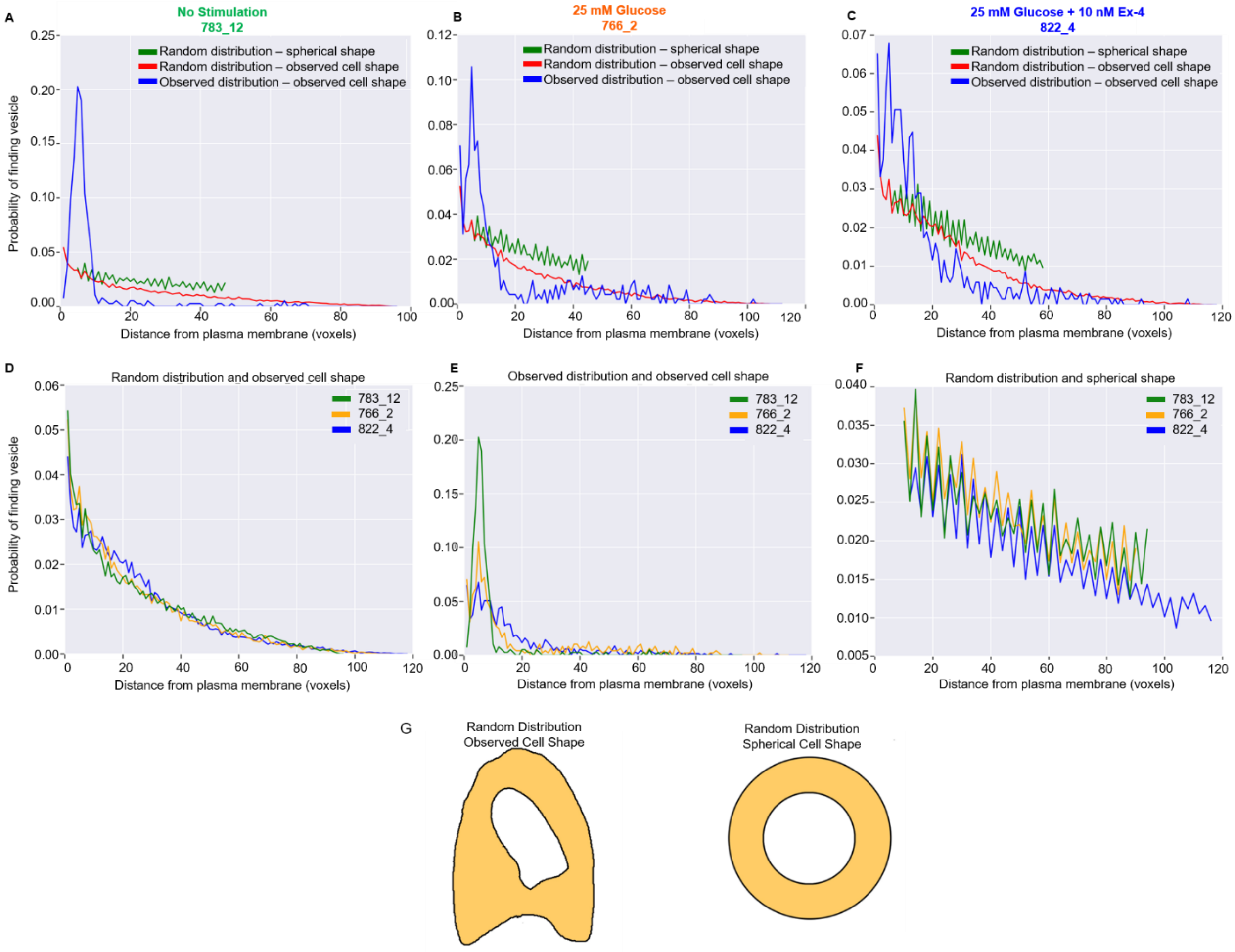

Fig. S4. Comparison of random versus observed distributions. Related to Fig. 3. (**A-C**) Plots of the probability of insulin vesicles being found within a given distance of the plasma membrane for a random distribution within a hypothetical sphere, a random distribution within the observed cell shape, and the observed distribution for representative cells. Comparison of the probability of insulin vesicles are found within a certain distance of the plasma membrane for (**D**) random distributions in observed cell shape, (**E**) observed distributions in observed cell shapes, and (**F**) random distributions in spherical cell shapes. (**G**) Cartoon representation of random distributions used in this figure. Yellow shaded region is the volume where granules were randomly distributed. The spherical cell and nucleus volumes were generated using volumes from the corresponding cell and nucleus masks of the cell. The two random distributions were slightly different because random distributions are proportional to the volume of the spherical shell along the distance to the plasma membrane. The difference between random distributions occurred only because the available

distance for the insulin vesicles was restricted to a certain value for spherical cell shapes, but for random distributions in observed cell shape, it was allowed extend as far as the observed distributions.

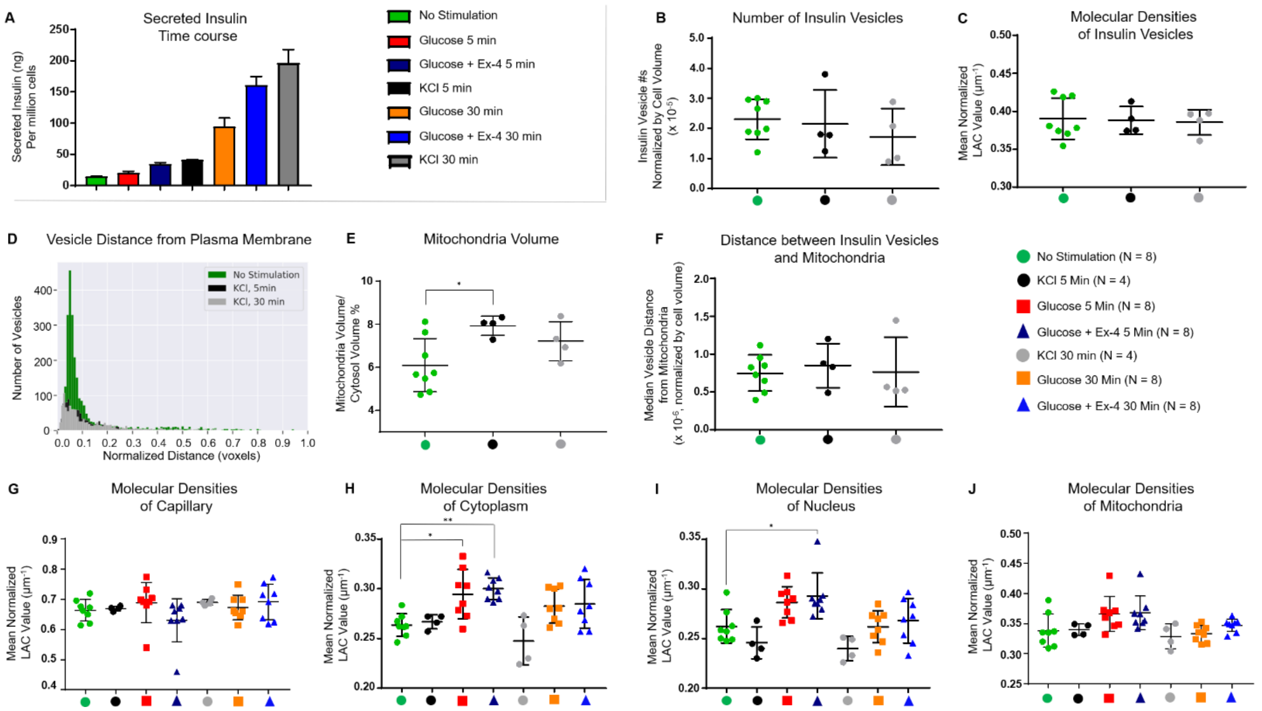

Fig. S5. Comparison of insulin secretion after stimulation at multiple timepoints and organelle molecular densities. Related to Fig. 3. (**A**) Plot of secreted insulin at several timepoints after stimulation. Performed in triplicate. (**B**) The number of insulin vesicles normalized by cell volume shows no statistical difference but a slight decrease in vesicle numbers in cells treated with KCl, corresponding to insulin secretion. (**C**) Mean molecular density (LAC value) of insulin vesicles in unstimulated and KCl treated cells shows no statistical differences. (**D**) Plot of the distribution distances of insulin vesicles from the plasma membrane. (**E**) Plot of the mitochondrial/cytosol volume ratio. Relative to the unstimulated condition, mitochondrial volume was larger for cells stimulated with KCl for 5-min (p = 0.0226). (**F**) Median mitochondria-insulin vesicle distance showing no statistical differences. (**G**) Mean LAC values of the glass capillary, showing no significant difference among conditions. (**H**) Mean LAC values of the cytoplasm (p = 0.0108 and 0.0020; * and **, respectively). (**I**) Mean nucleus LAC values (p = 0.0101). (**J**) Mean mitochondria LAC values, showing a trend for higher LAC values in cells treated for 5-min with glucose ± Ex-4. N values correspond to panels **B** – **J**. Statistical analysis performed by a Dunnett's comparison test.

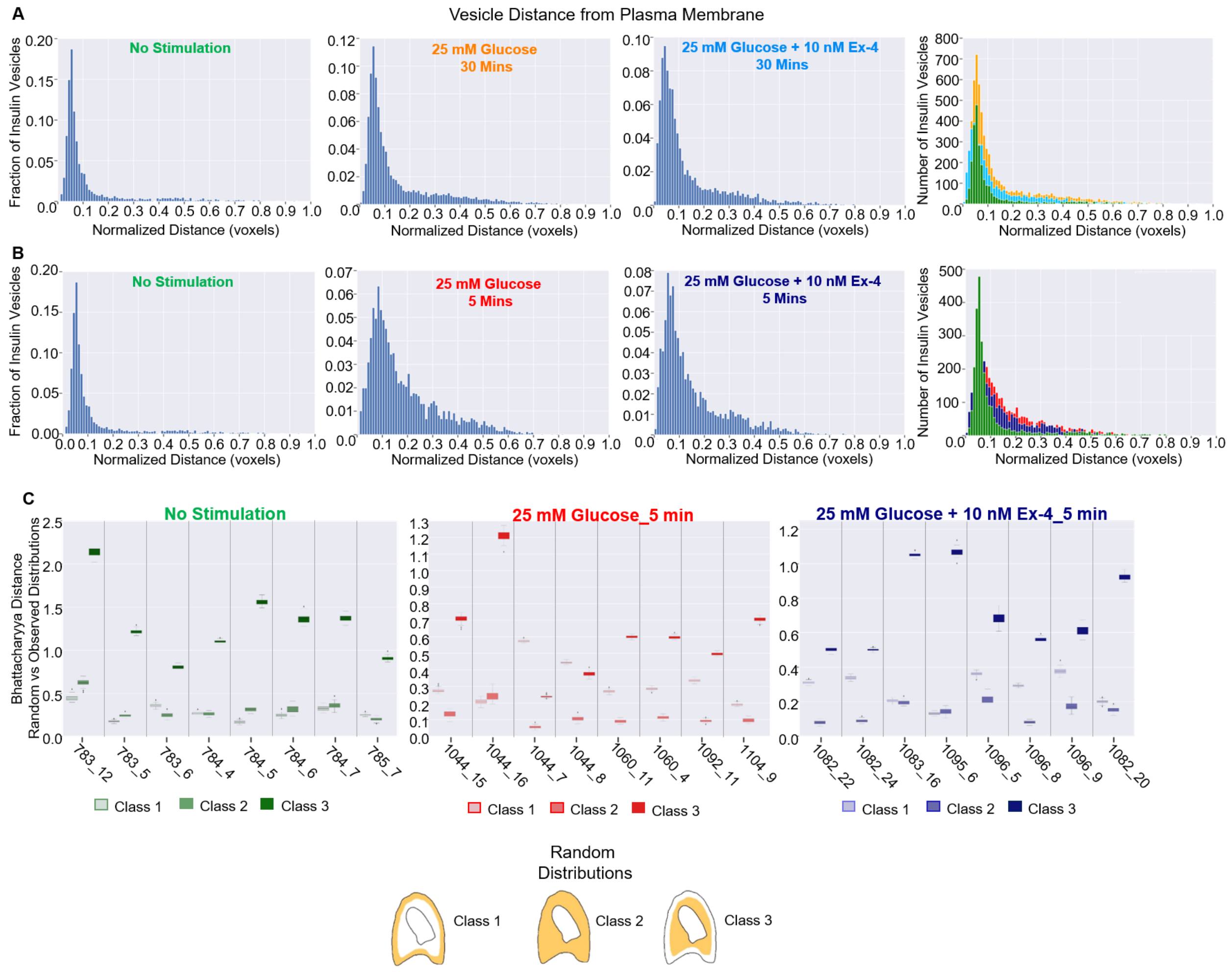

Fig. S6. Distributions of insulin vesicles at different timepoints after stimulation. Related to Fig. 3. Distribution of insulin vesicles from the plasma membrane for both stimulated conditions at 30-min (**A**) and 5-min (**B**) timepoints compared to the unstimulated condition. (**C**) Bhattacharyya Distance plots comparing random versus observed distribution of insulin vesicles. For each cell, the observed distribution of insulin vesicles was compared with 50 randomly distributed points shown here as scatter plots (left). Three classes of random distribution (right). The yellow highlighted region represents the volume of random distribution.

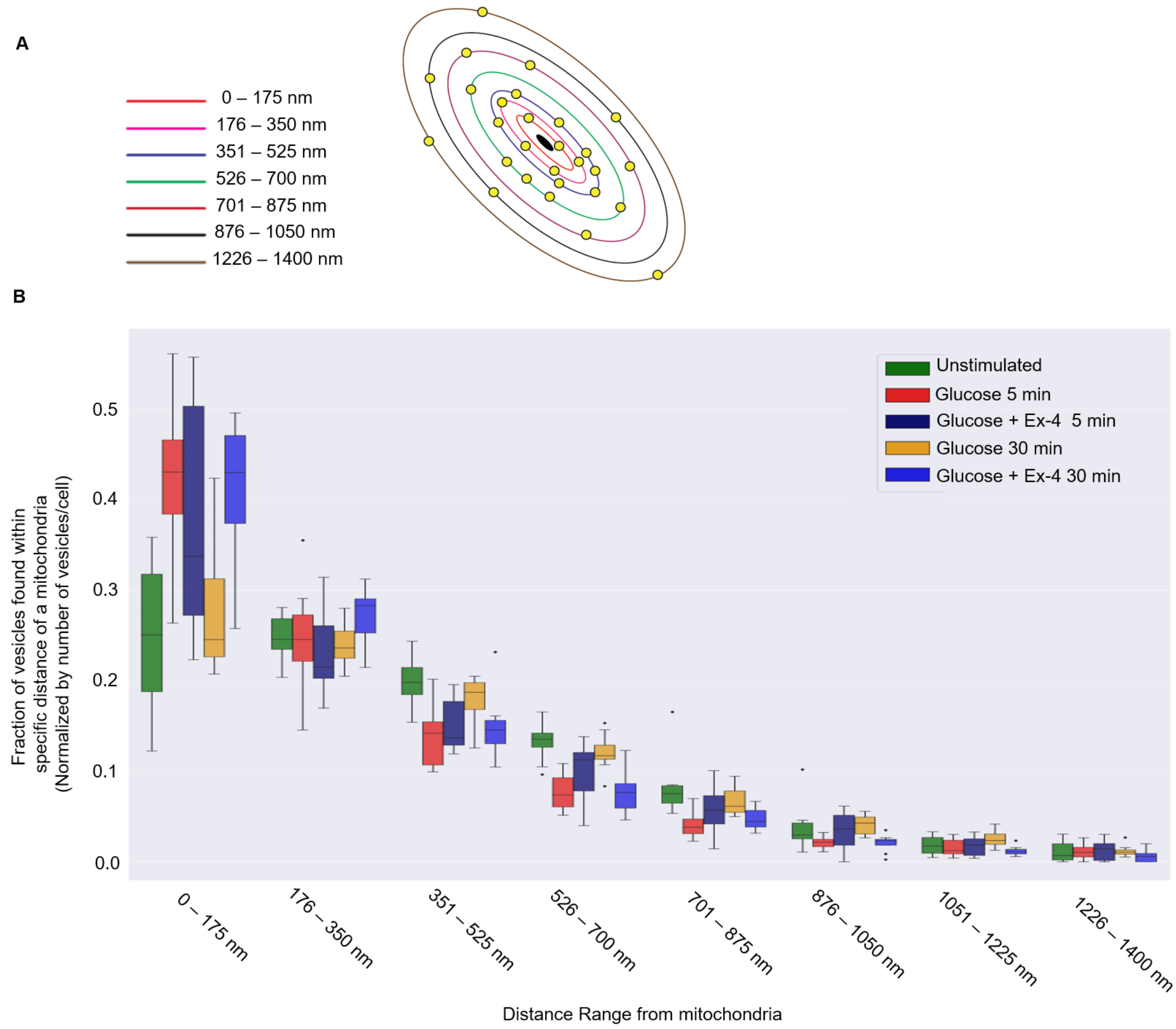

Fig. S7. Insulin vesicle distance to mitochondria. Related to Fig 3. (**A**) Cartoon representing the different distances from mitochondria investigated. The distance to the closest mitochondria was calculated for each insulin vesicle. The black oval in the center represents mitochondria. The yellow circles along the distance radii represent insulin vesicles. (**B**) Plot of the fraction of insulin vesicles found within a specific distance from the mitochondria.

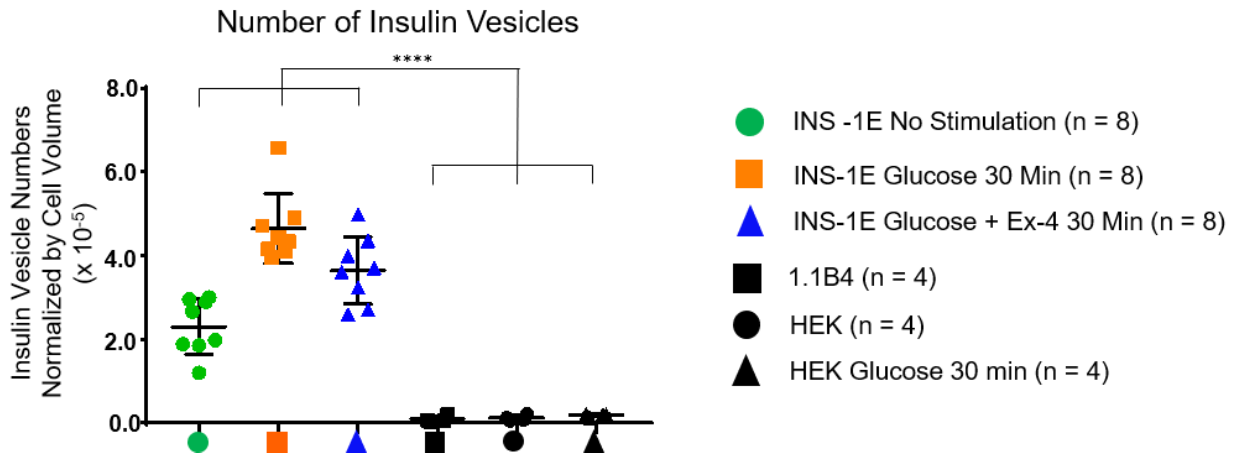

Fig. S8. Validation of insulin vesicle segmentation.

Number of insulin vesicles normalized by cell volume, showing statistical differences ($p < 0.0001$, ****; Tukey's multiple comparison test). All INS-1E conditions differed from each negative control.

Table S1. Reported values for stimulus conditions (excel sheet)

Table S2. Statistical analysis performed

Experiment	ANOVA	Post Hoc
**Fig. 1C** – Insulin secretion	$F_{(2,6)} = 108.3$, $p = 0.0001$, one-way ANOVA	Tukey's multiple comparison test: unstimulated and glucose, p = 0.0049; unstimulated and glucose + Ex-4, p < 0.0001; glucose and glucose + Ex-4 (p = 0.0002)
**Fig. 1D** – Mitochondria/Cytosol Ratio	$F_{(2,21)} = 3.909$, $p = 0.036$, one-way ANOVA	Dunnett's multiple comparison test, unstimulated and glucose + Ex-4 stimulated cells ($p = 0.0428$)
**Fig. 1E** – Insulin vesicle numbers	$F_{(2,21)} = 18.48$, $p < 0.0001$, one-way ANOVA	Tukey's multiple comparison test: unstimulated vs glucose and glucose + Ex-4, $p < 0.0001$ and $p = 0.0062$, respectively; glucose vs glucose + Ex-4, $p = 0.0436$
**Fig. 1F** – Insulin vesicle LAC values	$F_{(2,21)} = 4.75$, $p < 0.0199$, one-way ANOVA	Dunnett's multiple comparison test: unstimulated cells have a lower mean LAC of insulin vesicles compared to cells co-stimulated with glucose + Ex-4, $p = 0.0106$
**Fig. 3A** – Comparison of timepoint of stimulus on insulin vesicle numbers	$F_{(4,35)} = 12.8$, $p < 0.0001$, one-way ANOVA	Sidak's multiple comparison test: glucose for a timepoint of 5-min and 30-min, p = 0.0118; glucose + Ex-4, p = 0.0007; no difference between 5-min timepoint conditions and unstimulated
**Fig. 3B** – Comparison of timepoint of stimulus on insulin vesicle LAC	$F_{(4,35)} = 10.25$, $p < 0.0001$, one-way ANOVA	Sidak's multiple comparison test: glucose for a timepoint of 5-min vs 30-min, p = 0.0240; glucose + Ex-4 for timepoint of 5- vs 30-min, p = 0.0029; unstimulated vs glucose for 5-min and 30-min timepoints, $p \leq 0.001$ and 0.0001, respectively
**Fig. 3D** – Mitochondria volume	$F_{(4,35)} = 6.706$, $p = 0.0004$, one-way ANOVA	Holm-Sidak's multiple comparison test: unstimulated cells have a smaller volume than cells treated with glucose for 5-minutes, cells co-stimulated with glucose and Ex-4 for 5-min, and 30-min (p = 0.0252, 0.0007, 0.0325, respectively)
**Fig. 3E** – Mito-vesicle distance	$F_{(4,35)} = 7.495$, $p = 0.0002$, one-way ANOVA	Dunnett's multiple comparison test: significant difference between vesicle-mitochondria distances between unstimulated cells and cells stimulated with glucose 5-min ($p = 0.0099$), and glucose + Ex-4 for 5-min ($p = 0.0001$) and 30-min ($p = 0.0040$)
fig. S5E – KCl control cells mitochondrial volume	$F_{(2,13)} = 4.569$, $p = 0.0314$, one-way ANOVA	Dunnett's multiple comparisons test: significant difference between mitochondrial volume between unstimulated cells and cells stimulated with KCl 5-min ($p = 0.0226$). No significant difference between mitochondrial volumes in unstimulated cells and KCl 30-min cells ($p = 0.1754$).
fig. S5G – Comparison of capillary LAC of unstimulated INS-1E to glucose stimulated 5-min and 30-	$F_{(6,41)} = 1.257$, $p = 0.2984$, one-way ANOVA	No Difference in ANOVA

min, and co-stimulated with glucose + Ex-4 5-min and 30-min		
fig. S5H - Comparison of cytoplasm LAC of unstimulated INS-1E to glucose stimulated 5-min and 30-min, and co-stimulated with glucose + Ex-4 5-min and 30-min	$F_{(6,41)} = 5.858$, $p = 0.0002$, one-way ANOVA	Dunnett's multiple comparison test: There is a significant difference in cytoplasm LAC values between untreated cells and cells stimulated with glucose 5-min and co-stimulated with glucose + Ex-4 for 5-min (p = 0.0108 and 0.0020, respectively)
fig. S5I – Comparison of nucleus LAC of unstimulated INS-1E to glucose stimulated 5-min and 30-min, and co-stimulated with glucose + Ex-4 5-min and 30-min	$F_{(6,41)} = 6.517$, $p < 0.0001$, one-way ANOVA	Dunnett's multiple comparison test: there is a significant difference in nucleus LAC values between untreated cells and cells co-stimulated with glucose + Ex-4 5-min (p = 0.0101)
fig. S5J – Comparison of mitochondria LAC of unstimulated INS-1E to glucose stimulated 5-min and 30-min, and co-stimulated with glucose + Ex-4 5-min and 30-min	$F_{(6,41)} = 3.273$, $p = 0.0101$, one-way ANOVA	Dunnett's multiple comparison test: there was no significant difference in mitochondria LAC values between untreated and treated cells.
fig. S8 – Insulin vesicle number validation	$F_{(5,30)} = 54.61$, $p < 0.0001$, one-way ANOVA	Tukey's multiple comparison test: preselected pairs comparing INS-1E conditions to 1.1B4, HEK, and HEK Glucose negative controls where in each comparison $p < 0.0001$.
